# Retinochoroidal and Optic Nerve Head Microstructural and Microvascular Age-Related Changes in Healthy Eyes

**DOI:** 10.3390/diagnostics15050572

**Published:** 2025-02-27

**Authors:** Hamidu Hamisi Gobeka, Yiğit Şenol, Tolgonai Bektur Kyzy, İbrahim Ethem Ay, Mustafa Doğan

**Affiliations:** 1 Department of Ophthalmology, Faculty of Medicine, Afyonkarahisar Health Sciences University, Afyonkarahisar 03000, Türkiye; tolgonaibektur@gmail.com (T.B.K.); ibrahimethemay@windowslive.com (İ.E.A.); mustafadogan@yahoo.com (M.D.); 2 Department of Public Health, Afyonkarahisar Provincial Health Directorate, Afyonkarahisar 03000, Türkiye; dryigitsenol@hotmail.com

**Keywords:** aging, ganglion cell–inner plexiform layer thickness, OCTA, optic nerve head, retinochoroidal layer, SD-OCT

## Abstract

Background: To investigate the retinochoroidal and optic nerve head (ONH) microstructural and microvascular age-related changes in healthy subjects by examining the ganglion cell–inner plexiform layer thickness (GC-IPLT), vessel density (VD), and their ratio. Methods: In this cross-sectional study, 203 subjects (20–69 years old) were divided into five age groups: 20–29 (G1), 30–39 (G2), 40–49 (G3), 50–59 (G4), and 60–69 (G5) (G5). Following a thorough ophthalmological examination, enhanced depth imaging optical coherence tomography (EDI-OCT) scanning was performed along with OCT angiography (OCTA) in a 6 × 6 mm^2^ scanning area. Results: After adjusting for axial length the GC-IPLT varied significantly among groups, with thickness peaking in G3 (39.63 ± 1.14 µm) and then decreasing to the lowest in G5 (34.15 ± 5.93 µm) (*p* = 0.008). The whole, foveal, parafoveal, and perifoveal superficial and deep capillary plexus (SCP and DCP) VDs all varied significantly among groups, peaking in G2 and falling to their lowest in G5 (*p* < 0.05). No significant differences existed among groups regarding the GC-IPLT/whole SCP VD (*p* = 0.163) or GC-IPLT/whole DCP VD (*p* = 0.258) ratios. The foveal VDs in a 300-μm wide region surrounding the foveal avascular zone (FAZ) (FD-300) varied significantly among groups, peaking in G1 (57.06 ± 0.58) and dropping to its lowest in G5 (53.54 ± 0.59) (*p* < 0.05). The choriocapillaris flow differed significantly among groups, peaking in G1 (20.39 ± 0.15 mm^2^) and dropping to its lowest in G5 (19.24 ± 0.16 mm^2^) (*p* < 0.001). Conclusions: The retinochoroidal microstructure and microvasculature ratios display an inverted U-shaped pattern with age, which could be linked to a considerably decreased GC-IPLT versus capillary plexus VDs with age, notably in subjects in their sixties.

## 1. Introduction

As with the choroid, the retinal microstructure and microvasculature decrease with age [1,2]. Moreover, microstructural and microvascular morphological changes associated with retinal ganglion cells (RGCs) in the macula may essentially be employed as an indication of aging [3,4]. With the advancement of optical coherence tomography (OCT), it is now possible to detect and evaluate minor changes in retinal thickness as well as to quantitatively assess the efficacy of various treatment strategies. Essentially, OCT is a non-invasive optical imaging technique used in both clinical and research contexts to detect, track, and investigate various ocular disorders in vivo in either human or animal models. It generates high-resolution depth-resolved images encompassing several tissue layers without requiring contrast materials, and is used to capture in vivo cross-sectional structural and functional imaging of the retina [5,6]. A typical spectral domain (SD) OCT technique produces two quantitative RGC measurements: retinal nerve fiber layer thickness (RNFLT), which represents the number of RGC axons, and ganglion cell–inner plexiform layer thickness (GC-IPLT), which represents the number of RGC bodies and dendrites. In addition to measuring central macular thickness (CMT) and subfoveal choroidal thickness (SFCT), the SD-OCT’s great repeatability makes it useful for diagnosing optic nerve head (ONH) and retinochoroidal disorders [7].

Decreased GC-IPLT has been linked to aging, as have changes in the retinochoroidal microvascular morphology [8,9]. Likewise, there is a strong correlation between GC-IPLT and OCT angiography (OCTA) features [10]. The OCTA, a novel non-invasive imaging technology that enables high-resolution retinochoroidal microvascular visualization, has previously been used to assess the retinochoroidal microvascular morphological impairment in different ocular disorders [11,12,13]. It can be used to quantitatively evaluate microvasculature in each retinal layer and in the ONH, providing a comprehensive range of high precision quantitative parameters required for diagnosis and management of the retinochoroidal disorders [11,12,14]. Furthermore, this remarkable micrometer-level resolution, high sensitivity, and potentially wide field of view retinal imaging technology creates a blood flow map by comparing the decorrelation signal (differences in backscattered OCT signal intensity or amplitude) across sequential OCT B-scans obtained at the same cross-section [15,16].

Essentially, different conditions can have distinct impacts on the superficial and deep capillary plexus (SCP and DCP), implying that variables influencing normal retinochoroidal microvascular morphology in the capillary plexus could differ [17,18,19]. Additionally, the retinochoroidal atrophy may be accompanied by microvascular dysfunction, with poor perfusion potentially contributing to decreased thickness. In addition to the ONH, determining how the microstructural and microvascular morphology of the retinochoroidal layer, as well as their ratio, change with age could be clinically beneficial. Thus, our study aimed to determine the retinochoroidal and ONH microstructural and microvascular morphological age-related changes. By investigating the GC-IPLT, vessel densities (VDs), and their ratio, as well as the ONH global RNFLT and VDs, we hope to unravel a conundrum behind the precedence of potential physiological reduction of the retinochoroidal microstructure and microvascular morphology with age among healthy subjects.

## 2. Materials and Methods

### 2.1. Study Design and Subjects

In this single-center cross-sectional study, 203 healthy subjects (203 right eyes) ranging in age from 20 to 69 years old were recruited from hospital personnel and their spouses. They were divided into five age groups: 20–29 (G1), 30–39 (G2), 40–49 (G3), 50–59 (G4), and 60–69 (G5). The Afyonkarahisar Health Sciences University Institutional Review Board/Ethics Committee approved the study (Approval number: 2022/11, Ethics code: 2011-KAEK-2, Date: 2 September 2022), which adhered to the Helsinki Declaration tenets. Before inclusion, all subjects signed a written informed consent form.

Subjects did not have any systemic or ophthalmic diseases and were emmetrope with 20/20 uncorrected visual acuity. None of them regularly consumed caffeine-containing beverages or foods, nor did they smoke, consume alcohol or energy drinks, wear cosmetic or therapeutic contact lenses, or recently use eye lubricants. The following elements were listed as exclusion criteria: (i) a history of ocular diseases (lenticulo-corneal opacities, nystagmus, congenital or acquired retinal and neuro-ophthalmic diseases, glaucoma, and uveitis (anterior, intermediate, posterior, or panuveitis)), (ii) prior ocular trauma or surgery, including any anterior segment refractive surgery, (iii) pregnancy or breast-feeding, (iv) epilepsy, (v) migraine, (vi) systemic diseases (cardiovascular, endocrinology, hematologic, and/or neurologic diseases etc..) which could influence ocular microvasculature, (vii) a history of any chronic medications (antihistamines, decongestants, analgesics, sildenafil, etc.), (viii) an axial length (AXL) <21.00 or >26.00 mm, and (ix) an intraocular pressure (IOP) <10 or >21 mmHg.

### 2.2. Ophthalmological Assessment

The same senior ophthalmologist performed all tests under standard conditions. Initially, demographics were recorded, including age and gender. Afterwards a thorough ophthalmological examination was performed encompassing measurements of auto-refraction (Topcon Auto-refractometer model KM 8900, Tokyo, Japan), uncorrected visual acuity, IOPs (mmHg) (Goldmann; Haag-Streit AG, Köniz, Switzerland), and AXL (AL-Scan, Nidek CO., Gamagori, Japan), as well as anterior and posterior segment slit-lamp biomicroscopy (HaagStreit, Bern, Switzerland).

### 2.3. Enhanced Depth Imaging Optical Coherence Tomography Scanning

The Spectralis SD-OCT (Heidelberg Engineering, Heidelberg, Germany) can perform OCT scans at a rate of 20,000–40,000 A-scans/s [20]. It typically runs at 800–870 nm wavelengths, and its frame rate is determined by scan density (the number of A-scans within one B-scan) and camera read-out time. It features an axial resolution of 3.9 µm, a transverse resolution of 14 µm, and a scan depth of 1.9 mm. This technique fundamentally employs a dual-beam scanning system including a confocal scanning laser ophthalmoscopy reference beam for attaining reference scans for ocular movement tracking and a second beam for concurrently obtaining OCT images. The eye-tracking technology (TruTrack; Heidelberg Engineering) detects ocular movement and eliminates scans containing motion artifacts [21]; hence, a very efficient way of ensuring that the same areas of interest are scanned over time is created. This technique oversamples certain locations on OCT scans before comparing and combining them to decrease speckle (or random noise), improving visualization of structures of interest. Moreover, an enhanced depth imaging (EDI) improves visibility of the choroid and choroidoscleral interface using the SD system, which executes image averaging and sets the zero-delay line near the choroid [22].

In our study, the EDI-OCT scanning was carried out as previously described [22]. All procedures were conducted under mydriasis by one masked trained technician while a video image of the central retina was monitored. The foveal center was determined to be the point of greatest depression within a 500-µm radius [23]. The retinal thickness readings were obtained using a typical 25 × 25° raster scan methodology with horizontal scans separated by 240 μm. The CMT was identified as a distance between the vitreoretinal interface and the anterior surface of the retinal pigment epithelium (RPE) in the central area (1 mm radius) to represent an average CMT, which was automatically generated by the image-viewing software (Triton DRI OCT version 1.6.2.4, Heidelberg Eye Explorer version 6.0.9.0).

**Figure 1:**
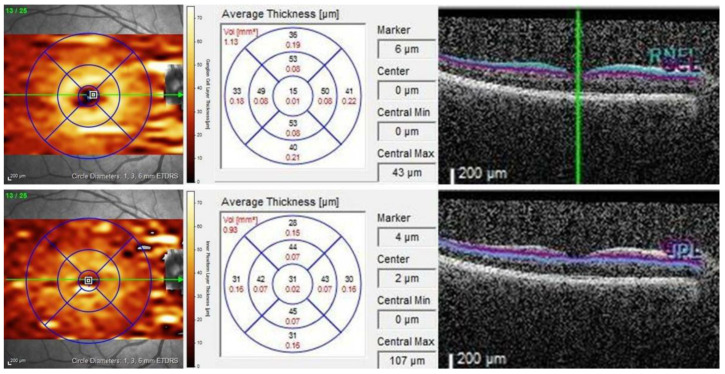
A representative scan employing Spectralis SD-OCT to show divisions of the segmented retinal layer. Retinal thickness analysis protocol displaying mean thicknesses for each subfield in the significance map: scan area of 6 × 6 mm^2^ divided into three concentric circles of 1 mm, 3 mm, and 6 mm diameters, respectively. The ganglion cell–inner plexiform layer is auto-segmented in horizontal B-scans and indicated by purple (denoting ganglion cell layer, top) and blue (denoting inner plexiform layer, below).

In addition, two senior ophthalmologists blindly classified and delineated all EDI-OCT images as per the proposed protocol. The SFCT was manually quantified as a vertical distance from the outer surface of a hyper-reflective line denoting the RPE and Bruch’s membrane complex to a hypo-reflective line denoting the sclero-choroidal interface centered on the fovea using a measuring tool with an integrated linear caliber. The average SFCT was calculated by averaging two blinded ophthalmologists’ SFCT measurements at the fovea as well as at 1500 and 3000 µm nasal and temporal distances from the foveal center. In addition, the device review software automatically segmented GCL and IPL with manual processing and correction as needed. The segmentation quality was thoroughly reviewed and any errors were corrected immediately. The GCL and IPL thickness measurements were recorded directly from the review software and the co-localized GCL and IPL thicknesses were added together manually to determine the GC-IPLT (Figure 1) [24].

### 2.4. Optical Coherence Tomography Angiography Acquisition

A single trained technician performed all OCTA (Optovue, Inc., Fremont, CA, USA) procedures in Angio Retina mode with a scanning area of 6 × 6 mm^2^. A scan quality indicator (SQI) was employed, which is a score of 1 to 10 issued by the device to OCTA images at the end of each acquisition by integrating signal intensity, ocular movements, and image focusing. Images with low-quality scans (SQI < 8) that exhibited fixation loss/incorrect foveal centration were discarded, as were those containing segmentation errors/motion artifacts, and the shots were repeated until the scan quality was satisfactory. The VDs and flow areas were automatically quantified using AngioVue Analytics (software version 2018.1.1.63), a quantitative analysis software.

#### 2.4.1. Capillary Plexus Vessel Density

**Figure 2:**
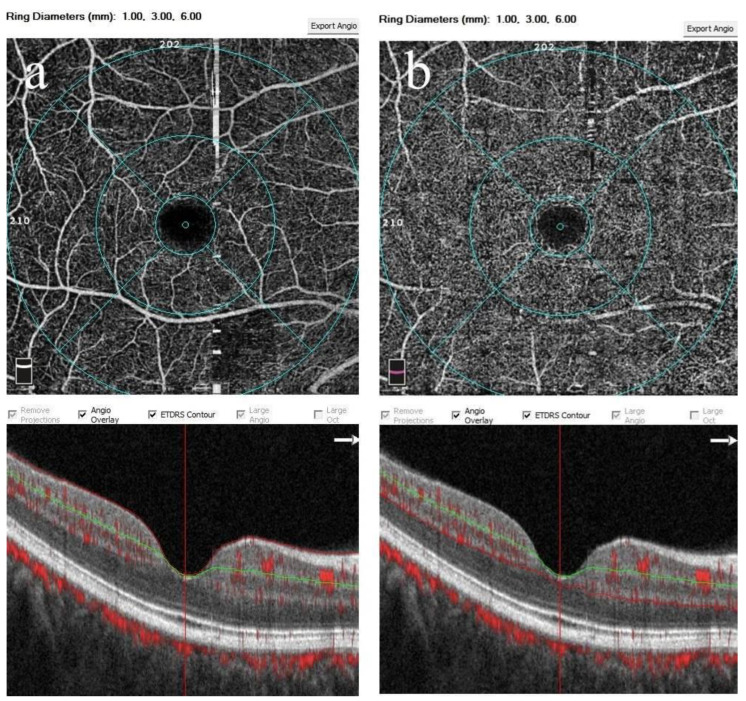
The 6 × 6 mm^2^ optical coherence tomography angiograms of a healthy subject’s right eye demonstrating the quantification of vessel densities in the whole, foveal, parafoveal, and perifoveal superficial (**a**) and deep (**b**) capillary plexus as depicted by Early Treatment of Diabetic Retinopathy Study (EDTRS) contours. The image below shows a cross-section through the macula centralis with normal foveal microstructural anatomy. Scan quality index: 9/10.

The percentage of pixels in a particular region covered by vessels and microvasculature was used to automatically quantify the VDs, which were determined using a 6 × 6 mm^2^ macular angiogram of the whole, foveal, parafoveal, and perifoveal SCP and DCP. While the SCP angiogram had an inner boundary 3 µm beneath the internal limiting membrane and an outer boundary 16 µm beneath the IPL, the DCP angiogram had an inner boundary 16 µm beneath the IPL and an outer boundary 70 µm beneath the IPL (Figure 2) [25].

#### 2.4.2. Foveal Avascular Zone and Capillary Flow Area

**Figure 3:**
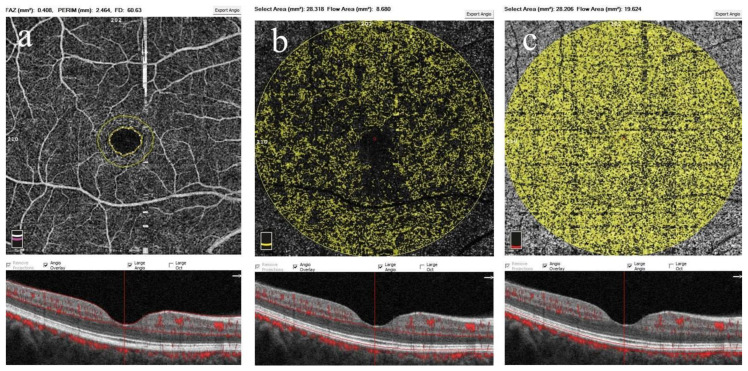
The 6 × 6 mm^2^ optical coherence tomography angiography en face slab of the right eye of a healthy subject, showing quantification of the foveal avascular zone parameters such as foveal avascular zone area and perimeter, as well as vessel densities in a 300 µm area around the foveal avascular zone (**a**) and capillary flow areas at the levels of the (**b**) outer retinal and (**c**) choriocapillaris layers. A cross-section through the macular center corresponding to the location of analysis is shown below. Scan quality index: 9/10.

The foveal avascular zone (FAZ) is typically a significant zone devoid of flow signal on an en face macular angiogram. This zone was analyzed automatically using FAZ mode software to determine parameters, including the FAZ area (mm^2^), FAZ perimeter (mm) and foveal VDs in a 300-μm wide region surrounding FAZ (FD-300) (%). Further, a maximum circular area that could be captured by the fovea-centered image section with a radius of 2.97 mm was manually created in the outer retinal and choriocapillaris layers. The outer retinal layer was located 70 μm beneath the IPL and 30 μm beneath the RPE. The AngioVue software calculated the outer retinal flow automatically as the percentage of vessel area covered in the selected area centered on the FAZ. The choriocapillaris layer was located between the RPE and deeper layer, with RPE offsets of 31 µm and 59 µm, respectively. Using Optovue software with a flow function, the choriocapillaris flow was calculated automatically by dividing choriocapillaris vessel areas by selected area (Figure 3) [26].

#### 2.4.3. Optic Nerve Head

The previously described Angio Analytics 2.0 (software version 2018.1.1.63) was used to quantify the ONH [27]. The VDs were quantified automatically as the proportion of the measured area occupied by flowing blood, which is identified as pixels with decorrelation values above the threshold level as determined by the split-spectrum amplitude-decorrelation angiography algorithm. In this context, the HD Angio Disc 4.5 mm mode was used to scan a 4.5 × 4.5 mm^2^ area around the ONH. This mode automatically produced the whole image VD (wiVD), as well as inside disc and peripapillary VDs. In this case, a 0.75 mm-wide elliptical annulus projecting from the ONH boundary was employed to define peripapillary area and calculate peripapillary VD. In addition, an optical disc 200 × 200 mode was used to obtain the ONH global RNFLT. Two senior ophthalmologists evaluated scans and exempted images with SQI < 8, poor clarity, residual motion artifacts visible as an anomalous vessel pattern or disk boundary on the en face angiogram, local weak signal, or serious segmentation failures.

### 2.5. Statistical Analysis

Statistical analysis was carried out using the Statistical Package for the Social Sciences (SPSS) 26.0 for Windows software (IBM Corp. in Armonk, NY, USA) [28]. Descriptive statistics employed percentages, frequencies, means, and standard deviations. The categorical data distribution was evaluated using the Chi-square test. The analysis of variance (ANOVA) and analysis of covariance (ANCOVA) tests were used to compare continuous data from more than three groups in accordance with the research questions. Age groups were used as the independent variables in the ANCOVA analysis, with AXL covariance and measurements taken as the dependent variables. Before conducting the ANOVA and ANCOVA analyses, the Kolmogorov–Smirnov test and graphical methods were used to assess the data’s fit to the normal distribution, which was found to be consistent. Before the ANCOVA analysis, the homogeneity of variances, linear relationship between the dependent variable and the covariate, and homogeneity of regression slopes were all evaluated and found to provide the assumptions for ANCOVA. The ANCOVA analysis yielded adjusted mean values. When ANCOVA and ANOVA analyses yielded statistically significant results, the Bonferroni correction was used in pairwise post hoc comparisons. A *p* < 0.05 was considered to be statistically significant.

## 3. Results

### 3.1. Demographic Features

G1-5 had 20.2%, 20.2%, 20.2%, 19.7%, and 19.7% of the total 203 subjects, respectively. The female-to-male ratios were comparable (*p* = 0.866). The IOP did not differ significantly among groups (*p* = 0.151). However, the AXL varied significantly among groups (*p* = 0.001), with the post hoc analysis revealing significant differences between G1 versus G5 (*p* = 0.045), G2 versus G4 (*p* = 0.025), and G2 versus G5 (*p* = 0.003) (Table 1).

### 3.2. Axial Length-Adjusted Microstructural Analysis

The CMT did not differ significantly among groups, despite being the lowest in G5 (*p* = 0.101). The GC-IPLT, on the other hand, varied significantly among groups, with the thickness peaking in G3 (39.63 ± 1.14 µm) and then decreasing to the lowest in G5 (34.15 ± 5.93 µm) (*p* = 0.008). The post hoc analysis revealed a significant difference between G3 versus G5 (*p* = 0.005). In addition, the SFCT differed significantly among groups (*p* = 0.001), with G1 (371.73 ± 7.76 µm) having the highest and G5 (326.55 ± 7.92 µm) having the lowest. In the post hoc analysis, there was a significant difference between G1 versus G2 (*p* = 0.016), G1 versus G3 (*p* = 0.018), and G1 versus G5 (*p* = 0.001) (Table 2).

### 3.3. Axial Length-Adjusted Microvascular Analysis

#### 3.3.1. Scan Quality Index

Table 3 contains detailed data on the comparison of the SQI and capillary plexus VDs by age group. As planned, all subjects had SQI > 8. Regardless, the SQI varied significantly across groups, peaking in G2 and then dropping to its lowest in G5 (*p* = 0.001). The post hoc analysis revealed a significant difference between G2 and G5 (*p* = 0.026).

#### 3.3.2. Capillary Plexus Vessel Density

The whole, foveal, parafoveal, and perifoveal SCP VDs varied significantly among groups, being highest in G2 and lowest in G5 (*p* < 0.05, for all). The post hoc analysis revealed significant differences in: (a) the whole SCP VDs between G2 versus G5 (*p* < 0.001), G3 versus G5 (*p* = 0.028), as well as G4 versus G5 (*p* = 0.017); (b) the foveal and perifoveal SCP VDs between G2 and G5 (*p* = 0.042 and 0.005, respectively); and (c) the parafoveal SCP VDs between G5 versus all other age groups (*p* < 0.05 for all).

Likewise, the whole, foveal, parafoveal, and perifoveal DCP VDs varied significantly among groups, being highest in G2 and lowest in G5 (*p* < 0.05, for all). The post hoc analysis revealed significant differences in: (a) the whole and foveal DCP VDs between G5 versus all other groups (*p* < 0.05 for all); (b) the parafoveal DCP VDs between G1 versus G4 (*p* = 0.040) and G2 versus G4 (*p* = 0.018), as well as G5 versus all other age groups (*p* < 0.05, for all); and (c) the perifoveal DCP VDs between G2 versus G4 (*p* = 0.015) as well as G5 versus all other age groups (*p* < 0.05, for all).

#### 3.3.3. Ratio Analyses

No significant difference existed among groups regarding the GC-IPLT/whole SCP VD and GC-IPLT/whole DCP VD ratios (*p* = 0.163 and 0.258, respectively) (Table 4).

#### 3.3.4. Foveal Avascular Zone and Capillary Flow Area

Neither the FAZ area (*p* = 0.299) nor the FAZ perimeter (*p* = 0.155) differed significantly among groups. However, the FD-300, like the capillary plexus VDs, varied significantly among groups, being highest in G1 (57.06 ± 0.58) and lowest in G5 (53.54 ± 0.59) (*p* < 0.05). The post hoc analysis revealed significant differences between G1 versus G5 (*p* < 0.001), G2 versus G5 (*p* = 0.011), and G3 versus G5 (*p* = 0.037).

While the outer retinal flow did not differ significantly among groups (*p* = 0.228), the choriocapillaris flow varied significantly, being highest in G1 (20.39 ± 0.15 mm^2^) and lowest in G5 (19.24 ± 0.16 mm^2^) (*p* < 0.001). The post hoc analysis revealed significant differences in the choriocapillaris flow between G1 versus all other age groups (*p* < 0.05, for all) (Table 5).

### 3.4. Optic Nerve Head Analysis

As displayed in Table 6, none of the ONH global RNFLT (*p* = 0.315), wiVD (*p* = 0.188), inside disc (*p* = 0.204), or peripapillary (*p* = 0.279) VDs differed significantly among groups, nor did the cup–disc ratio (*p* = 0.911).

## 4. Discussion

As the elderly population increases, vision and cognitive functioning decrease, posing a significant threat to public health [29]. Aging has been related to retinal neurodegeneration based on the mean thickness of the neuronal layers, including the RNFLT and GC-IPLT [30]. Because the retina is an extension of the brain, it provides an unparalleled opportunity to investigate the impact of aging on the central nervous system as well as the physiological and pathological systems associated with aging [31]. It appears that retinochoroidal disorders disproportionately affect the elderly. As a result, detecting genuine disorders requires identifying typical age-related retinochoroidal microstructural and microvascular morphological changes. In this perspective, we used SD-OCT and OCTA to investigate these age-related changes among healthy subjects after adjusting for the AXL.

Essentially, close monitoring of the RGCs and their neural processes, such as axons and dendrites, is possible by measuring the thickness of the peripapillary RNFL, neuroretinal rim at the ONH, or inner retinal layers in the macula [30,32]. The GC-IPLT may be as accurate as RNFLT or ONH parameters in detecting likely perimetric glaucoma [33,34]. In addition, the GC-IPLT decreases in several ocular disorders, suggesting that it could be a valuable biomarker in the diagnosis and treatment of certain ocular disorders. For instance, the GC-IPLT reduction rates in high myopes in their fifties [9] and eyes with diabetic retinopathy [8] have been found to be −0.81 m/year and −0.987 m/year, respectively. Hence, to accurately analyze such changes in GC-IPLT, determining its age-related physiological trend appears clinically worthwhile. We found that the older the healthy subjects were, the higher the overall decline in microstructural parameters, with this decrease being significant in GC-IPLT and SFCT. In all, these findings are mostly consistent with past research, which has primarily reported on age-related GC-IPLT changes [2,8,9,34]. Such physiological alterations appear to be essential in deciphering the GC-IPLT variations in the elderly.

Globe elongation has been linked to a decreased retinal microvasculature in myopic eyes [35]. In addition, the AXL has been found to be positively correlated with the SCP VD/DCP VD ratio. This condition implies that the DCP VD reduction is greater than the SCP VD decrease as the AXL length increases [36,37,38]. We revealed significant differences in AXL among groups, with post hoc analysis revealing significant differences between younger and elderly subjects. As a result, both the SCP and the DCP VDs were revealed to be lower in younger subjects. Because the DCP has more microvascular density than the SCP, it may be more responsive to changes in globe elongation. As the AXL increases, the DCP VDs may decrease more than the SCP VDs [25]. However, the AXL-related effect would be restricted. Thus, in addition to excluding eyes with AXLs > 26.0 mm, we used age groups as independent variables in the ANCOVA analysis, with the AXL covariance and measurements as dependent variables, in order to obtain adjusted mean values.

The OCTA image quality determines VD quantification and reliability. Lower SCP and DCP VDs have been reported to be significantly associated with decreased SQI, which greatly influenced retinal VDs [37]. However, as the SQI increased, so did the OCTA measures [8]. Furthermore, in angiograms with >25 dB, the SCP VD/DCP VD ratio has been found to positively correlate with OCTA quality [36]. Despite a cut-off SQI of more than eight, our analysis revealed considerably different SQIs among age groups, with subjects in their thirties having the greatest and subjects in their sixties having the lowest. Thus, when comparing retinochoroidal microvascular morphology, the quality of the OCTA angiograms appears to be essential.

Findings suggest that the parafoveal VDs decrease by 0.116% [2], and 0.4% [1] per year in healthy subjects, respectively. OCTA parameter changes that go beyond these physiological changes may be an indication of a specific disease; consequently, examining the patterns of age-related physiological changes becomes critical. As a matter of fact, age has a significant impact on the majority of the peripapillary and macular VDs [2]. While the SCP and DCP VDs have a negative correlation with age, the SCP VD/DCP VD ratio is significantly correlated with age, presumably due to the fact that the capillary plexus is in distinct regions [36]. In our study, we discovered that after adjusting for the AXL, older subjects had greater the overall microvascular morphological decreases in the SCP and DCP VDs in all macular regions. The same was true for the FD-300, which was lowest in subjects in their sixties, accompanied by a non-significant age-related increase in the FAZ area and perimeters. In addition, the choriocapillaris flow declined with age, with the lowest values found in patients in their sixties.

In general, aging was associated with decreased microvascular morphology in the retinochoroidal layer in our study. This age-related physiological decrease among healthy subjects could be related to decreased cerebral blood flow, which has been reported to decrease by 0.50% per year with age [39] A relatively significant decrease in the capillary plexus VDs, particularly in subjects in their sixties, could indicate that the retinochoroidal microvascular morphology in the elderly is not constant. Moreover, the thickness of the inner retina (where SCP is found), which includes the RNFL, GCL, and IPL, decreased with age [11,40]. A relationship between inner retinal layer thickness and SCP VDs has been established; consequently, SCP changes may signify a thinning inner retina that diminishes with age [41]. The DCP, on the other hand, is found on the INL and has a rather constant thickness across time. As a result, the SCP would be more susceptible to VD changes with age than the DCP [36].

Because the location, size, and morphology of the SCP and DCP differ, various factors could impact them differently [37]. In normal eyes, the SCP VD/DCP VD ratio correlates strongly with age, AXL, and OCTA quality [36]. However, we found no significant age-related differences in the retinochoroidal microstructure or microvasculature ratios expressed as GC-IPLT/whole SCP VD and GC-IPLT/whole DCP VD ratios. Despite this, these ratios followed an inverted U-shaped pattern, with middle-aged subjects, i.e., those in their forties and fifties, having higher ratios than other age groups. Although both microstructural and microvascular morphological parameters were higher, this indicated that middle-aged subjects were associated with relatively higher GC-IPLT than capillary plexus VDs, which decreased dramatically in subjects in their sixties. In other words, while both parameters decreased with age, this condition could be associated with the age-related marked reduction in the GC-IPLT versus capillary plexus VDs, particularly in subjects in their sixties. Most importantly, the SCP and DCP VDs constitute the microvasculature from the internal limiting membrane to the IPL of the macular region, which contains the GC-IPL. Thus, the capillary plexus VDs and GC-IPLT are inextricably linked. Anatomical issues such as chorioretinal atrophy, a type of chorioretinal degeneration with a relatively well-defined boundary of the posterior pole, can actually have a negative impact on the retinochoroidal microvascular morphology. This could lead to decreased blood flow and substantial retinal thinning, and vice versa [42]. Considering the decreases in both GC-IPLT and retinochoroidal VDs, the lower GC-IPL/VD ratios in both SCP and DCP reflect a breakdown in the balance of anatomical microstructure with retinal microperfusion. This balance may have been preserved by relatively prominent alterations in the microstructural anatomy, particularly in middle-aged subjects rather than those in their sixties, when this disparity occurred markedly.

More specifically, variation in age-related retinochoroidal microstructure and microvasculature ratios signifies that physiological changes in the retinochoroidal microstructural anatomy were more pronounced in middle-aged subjects when compared to microvascular changes in subjects in their sixties. Despite concurrent retinochoroidal microstructure and microvasculature decrement, it appears that age-related inner retinal thinning occurs before changes in the retinochoroidal microvascular system. Actually, it is assumed that a decrease in the retinochoroidal microstructural thickness occurs concurrently with or as a result of an otherwise clinically unnoticed decline in physiological retinochoroidal microperfusion with age, with the former being more likely. More long-term studies are needed to pinpoint the exact relationship. Additionally, because the SCP and DCP VDs for each OCTA device differ, different OCTA devices may display different ratios [43]. However, the trend appears to be continuing, and the current study findings appear to be applicable in clinical settings and prospective OCTA research.

Moreover, the RNFLT and GC-IPLT have been found to decrease with age in both diseased and healthy eyes [40]. Likewise, we found potentially diminished ONH microstructural and microvascular morphology with age progression. Changes in the ONH global RNFLT, as well as the wiVD, inside disc VDs, or peripapillary VDs, tended to decrease with age, with subjects in their sixties having the lowest level. A similar mechanism could apply to the ONH OCTA findings, as it does to retinochoroidal OCTA findings.

Our study has a number of limitations. The cross-sectional study design precluded investigating the longitudinal relationship of age-related physiological changes in the retinochoroidal and ONH microstructure and microvascular morphology. Nonetheless, consistent rates of age-related thinning in GCLT have been reported in longitudinal and cross-sectional studies [44]. The majority of the healthy subjects were from Türkiye’s Aegean region. These subjects were distinguished by their predominantly middle socio-economic status, and they were recruited as volunteers from hospital personnel and their spouses, which may limit generalizability to a broader age range. Subjects beyond the age of 70 were not recruited due to inadequate visual acuity or poor OCTA quality. We concentrated on the visualization of age-related changes in intraretinal layers. In healthy emmetrope subjects, the GC-IPLT thickness changed dramatically with age; however, more research is needed to determine whether such changes in thickness have any effect on vision. Because eyes with AXL > 26.0 mm were exempted, more research, including high myopia, is needed to more accurately determine the relationship between the AXL and OCTA parameters. Furthermore, the body mass index was not calculated, which could have an impact on the microvascular morphology [45].

Despite its limitations, our study has several advantages. It could be the first study to quantitatively analyze age-related changes in retinochoroidal and ONH anatomical microstructure and microvascular morphology, as well as the first to investigate the associated ratio trend across different age groups of a relatively large number of healthy subjects. Only high-quality OCT angiograms with SQI >8 were considered, allowing for more accurate analyses. On top of this, the analyses were AXL-adjusted to exclude any potential influence of the AXL on the OCT and OCTA findings.

## 5. Conclusions

Apart from the SFCT, we found a substantial link between the GC-IPLT and aging, which is supported by prior research. These physiological changes appear to be critical in deciphering GC-IPLT differences in the elderly. In addition, we noted that older subjects had greater overall microvascular morphological decrease in the capillary plexus VDs as well as in the FD-300 and choriocapillaris flow, perhaps due to decreased cerebral blood flow. Indeed, it is possible to propose non-constant retinochoroidal microvascular morphology in the elderly. The retinochoroidal microstructure and microvasculature ratios showed an inverted U-shaped pattern with age, which could be associated with a significantly decreased GC-IPLT versus capillary plexus VDs with age, particularly in subjects in their sixties. Changes in the ONH global RNFLT and VDs tended to decrease with age, reflecting a process similar to the retinochoroidal OCTA findings.

## Figures and Tables

**Table 1:** Demographic features of the study subjects (*n* = 203).

Parameters	Age Groups (Mean ± Standard Deviation)					*p* Value
	G1	G2	G3	G4	G5	
Age (years)	24.93 ± 2.82	33.95 ± 2.65	43.29 ± 2.57	53.15 ± 2.86	63.55 ± 2.80	
Female [*n* (%)]/Male [*n* (%)]	24 (58.5)/17 (41.5)	22 (53.7)/19 (46.3)	21 (51.2)/20 (48.8)	20 (50.0)/20 (50.0)	24 (60.0)/16 (40.0)	0.866
IOP (mmHg)	14.90 ± 2.60	16.43 ± 3.32	15.37 ± 3.10	15.30 ± 3.03	15.00 ± 2.91	0.151
AXL (mm)	22.22 ± 1.03	22.03 ± 1.01	22.55 ± 1.06	22.67 ± 0.75	22.80 ± 0.84	0.001 ***

IOP = Intraocular pressure, mmHg = millimeter of mercury, AXL = Axial length, mm = millimeter, G1 = Subjects aged 20–29 years, G2 = Subjects aged 30–39 years, G3 = Subjects aged 40–49 years, G4 = Subjects aged 50–59 years, and G5 = Subjects aged 60–69 years, *n* = Number of subjects, *** *p* < 0.05.

**Table 2:** Comparative analysis of microstructural parameters among groups (*n* = 203).

Parameters	Data Analyses	Age Groups	Observed Mean ± SD		Adjusted Mean ± SE		Post-Hoc Analysis	
CMT (µm)	Descriptive statistics	G1	263.12 ± 17.21		262.96 ± 2.59			
		G2	264.51 ± 16.22		264.22 ± 2.62			
		G3	268.37 ± 17.70		268.44 ± 2.58			
		G4	267.75 ± 18.07		267.90 ± 2.62			
		G5	259.40 ± 12.32		259.65 ± 2.64			
			SS	DF	MS	F	*p* value	Partial η^2^
	Ancova results	AXL	88.712	1	88.712	0.327	0.568	0.002
		Age group	2135.394	4	533.848	1.968	0.101	0.038
		Error	53,452.534	197	271.333			
GC-IPLT (µm)	Descriptive statistics	G1	36.90 ± 7.22		37.09 ± 1.15		G3 > G5(0.005)	
		G2	37.76 ± 6.84		38.09 ± 1.16			
		G3	39.71 ± 8.38		39.63 ± 1.14			
		G4	38.58 ± 7.95		38.41 ± 1.16			
		G5	34.15 ± 5.93		33.87 ± 1.17			
			SS	DF	MS	F	*p* value	Partial η^2^
	Ancova results	AXL	111.400	1	111.400	2.091	0.150	0.011
		Age Group	757.223	4	189.306	3.554	0.008 **	0.067
		Error	10,493.133	197	53.265			
SFCT (µm)	Descriptive statistics	G1	371.51 ± 50.67		371.73 ± 7.76		G1 > G2(0.016) G1 > G3(0.018) G1 > G5(0.001)	
		G2	336.37 ± 52.29		336.75 ± 7.87			
		G3	337.02 ± 47.44		336.93 ± 7.72			
		G4	341.98 ± 45.11		341.78 ± 7.85			
		G5	326.88 ± 50.52		326.55 ± 7.92			
			SS	DF	MS	F	*p* value	Partial η^2^
	Ancova results	AXL	149.630	1	149.630	0.061	0.805	0.000
		Age Group	46,841.925	4	11,710.481	4.798	0.001 **	0.089
		Error	480,806.452	197	2440.642			

CMT = Central macular thickness, GC-IPLT = Ganglion cell-inner plexiform layer thickness, SFCT = Subfoveal choroidal thickness, SD = Standard deviation, SE = Standard error, AXL = Axial length, G1 = Subjects aged 20–29 years, G2 = subjects aged 30–39 years, G3 = Subjects aged 40–49 years, G4 = Subjects aged 50–59 years, and G5 = Subjects aged 60–69 years, µm = Micrometer, η^2^ = Eta Squared, *n* = Number of subjects, ** *p* < 0.01.

**Table 3:** Age-group comparison of SQI and capillary plexus VDs (*n* = 203).

Parameters	Data Analyses	Age Groups	Observed Mean ± SD		Adjusted Mean ± SE		Post-Hoc Analysis	
SQI	Descriptive statistics	G1	8.49 ± 0.68		8.50 ± 0.12		G2 > G5 (0.026)	
		G2	8.56 ± 0.67		8.57 ± 0.12			
		G3	8.49 ± 0.78		8.49 ± 0.12			
		G4	8.20 ± 0.82		8.19 ± 0.12			
		G5	8.05 ± 0.85		8.04 ± 0.12			
			SS	DF	MS	F	*p* value	Partial η^2^
	Ancova results	AXL (mm)	0.171	1	0.171	0.293	0.589	0.001
		Age group	7.934	4	1.984	3.406	0.010 **	0.065
		Error	114.715	197	0.582			
Whole SCP VDs (%)	Descriptive statistics	G1	52.11 ± 2.83		52.06 ± 0.47		G2 > G5 (<0.001) G3 > G5 (0.028) G4 > G5 (0.017)	
		G2	53.72 ± 3.01		53.63 ± 0.48			
		G3	52.27 ± 3.10		52.29 ± 0.47			
		G4	52.35 ± 2.58		52.40 ± 0.48			
		G5	50.20 ± 3.40		50.27 ± 0.48			
			SS	DF	MS	F	*p* value	Partial η^2^
	Ancova results	AXL (mm)	8.067	1	8.067	0.899	0.344	0.005
		Age group	223.013	4	55.753	6.211	<0.001 ***	0.112
		Error	1768.342	197	8.976			
Foveal SCP VDs (%)	Descriptive statistics	G1	23.26 ± 5.39		23.16 ± 1.17		G2 > G5 (0.042)	
		G2	24.35 ± 9.18		24.18 ± 1.18			
		G3	23.42 ± 8.16		23.46 ± 1.16			
		G4	21.65 ± 7.07		21.74 ± 1.18			
		G5	19.09 ± 6.72		19.24 ± 1.19			
			SS	DF	MS	F	*p* value	Partial η^2^
	Ancova results	AXL (mm)	28.725	1	28.725	0.520	0.472	0.003
		Age group	581.764	4	145.441	2.635	0.035	0.051
		Error	10,872.803	197	55.192			
Parafoveal SCP VDs (%)	Descriptive statistics	G1	54.86 ± 2.31		54.85 ± 0.57		G1 > G5 (0.021) G2 > G5 (<0.001) G3 > G5 (0.012) G4 > G5 (0.021)	
		G2	55.84 ± 3.31		55.83 ± 0.57			
		G3	54.68 ± 4.03		54.68 ± 0.56			
		G4	54.52 ± 3.30		54.52 ± 0.57			
		G5	52.64 ± 4.59		52.65 ± 0.58			
			SS	DF	MS	F	*p* value	Partial η^2^
	Ancova results	AXL (mm)	0.137	1	0.137	0.011	0.918	0.000
		Age group	201.982	4	50.495	3.905	0.004 **	0.073
		Error	2547.338	197	12.931			
Perifoveal SCP VDs (%)	Descriptive statistics	G1	52.17 ± 5.36		52.07 ± 0.57		G2 > G5 (0.005)	
		G2	53.93 ± 2.90		53.75 ± 0.58			
		G3	52.83 ± 2.97		52.88 ± 0.56			
		G4	52.48 ± 2.80		52.57 ± 0.57			
		G5	50.68 ± 3.40		50.83 ± 0.58			
			SS	DF	MS	F	*p* value	Partial η^2^
	Ancova results	AXL (mm)	33.620	1	33.620	2.584	0.110	0.013
		Age group	179.738	4	44.934	3.453	0.009 **	0.066
		Error	2563.626	197	13.013			
Whole	Descriptive statistics	G1	56.07 ± 4.42		56.08 ± 0.82		G1 > G5 (<0.001) G2 > G5 (<0.001) G3 > G5 (<0.001) G4 > G5 (0.004)	
		G2	57.29 ± 4.98		57.30 ± 0.83			
		G3	56.67 ± 5.94		56.67 ± 0.82			
		G4	54.25 ± 5.38		54.24 ± 0.83			
		G5	50.06 ± 5.26		50.04 ± 0.84			
			SS	DF	MS	F	*p* value	Partial η^2^
	Ancova results	AXL (mm)	0.255	1	0.255	0.009	0.923	0.000
		Age group	1308.388	4	327.097	11.951	0.000 ***	0.195
		Error	5392.001	197	27.371			
Foveal DCP VDs (%)	Descriptive statistics	G1	39.82 ± 5.95		39.88 ± 1.12		G1 > G5 (0.001) G2 > G5 (<0.001) G3 > G5 (<0.001) G4 > G5 (0.030)	
		G2	40.73 ± 6.02		40.84 ± 1.14			
		G3	40.65 ± 9.07		40.63 ± 1.12			
		G4	37.82 ± 8.18		37.76 ± 1.13			
		G5	34.36 ± 5.72		34.26 ± 1,14			
			SS	DF	MS	F	*p* value	Partial η^2^
	Ancova results	AXL (mm)	12.223	1	12.223	0.240	0.625	0.001
		Age group	1157.237	4	289.309	5.685	0.000 ***	0.103
		Error	10,024.935	197	50.888			
Parafoveal DCP VDs (%)	Descriptive statistics	G1	58.64 ± 3.84		58.66 ± 0.68		G1 > G4 (0.040) G1 > G5 (<0.001) G2 > G4 (0.018) G2 > G5 (<0.001) G3 > G5 (<0.001) G4 > G5 (0.023)	
		G2	58.96 ± 4.68		58.10 ± 0.69			
		G3	58.41 ± 4.67		58.40 ± 0.67			
		G4	56.69 ± 4.06		56.67 ± 0.68			
		G5	54.48 ± 4.13		54.45 ± 0.69			
			SS	DF	MS	F	*p* value	Partial η^2^
	Ancova results	AXL (mm)	1.642	1	1.642	0.089	0.766	0.000
		Age group	544.385	4	136.096	7.346	0.000 ***	0.130
		Error	3649.721	197	18.527			
Perifoveal DCP VDs (%)	Descriptive statistics	G1	57.07 ± 5.73		57.06 ± 0.91		G4 > G5(0.001)	
		G2	59.31 ± 4.98		59.29 ± 0.92		G1 > G5(<0.001)	
		G3	58.41 ± 6.28		58.42 ± 0.91		G2 > G4(0.015)	
		G4	56.05 ± 5.96		56.06 ± 0.92		G2 > G5(<0.001)	
		G5	51.46 ± 5.88		51.48 ± 0.93		G3 > G5(<0.001)	
			SS	DF	MS	F	*p* value	Partial η^2^
	Ancova results	AXL (mm)	0.404	1	0.404	0.012	0.913	0.000
		Age group	1424.797	4	356.199	10.610	0.000 ***	0.177
		Error	6613.737	197	33.572			

SQI = Signal quality index, VDs = Vessel densities, SD = Standard deviation, SE = Standard error, AXL = Axial length, mm = millimeter, SCP = Superficial capillary plexus, DCP = Deep capillary plexus, % = Percentage, *n* = Number of subjects, ** *p* < 0.01, *** *p* < 0.001.

**Table 4:** Age-group comparison of GC-IPLT/whole SCP VD and GC-IPLT/whole DCP VD ratios (*n* = 203).

Ratios	Data Analyses	Age Groups	Observed Mean ± SD		Adjusted Mean ± SE		Post-Hoc Analysis	
GC-IPLT/whole SCP VDs (μm/%)	Descriptive statistics	G1	0.70 ± 0.14		0.71 ± 0.02			
		G2	0.70 ± 0.14		0.71 ± 0.02			
		G3	0.76 ± 0.17		0.76 ± 0.02			
		G4	0.74 ± 0.16		0.73 ± 0.02			
		G5	0.69 ± 0.15		0.68 ± 0.02			
			SS	DF	MS	F	*p* value	Partial η^2^
	Ancova results	AXL (mm)	0.057	1	0.057	2.521	0.114	0.013
		Age group	0.149	4	0.037	1.652	0.163	0.032
		Error	4.444	197	0.023			
GC-IPLT/whole DCP VDs (μm/%)	Descriptive statistics	G1	0.66 ± 0.14		0.66 ± 0.02			
		G2	0.67 ± 0.14		0.67 ± 0.03			
		G3	0.73 ± 0.18		0.73 ± 0.02			
		G4	0.72 ± 0.17		0.72 ± 0.03			
		G5	0.69 ± 0.15		0.69 ± 0.03			
			SS	DF	MS	F	*p* value	Partial η^2^
	Ancova results	AXL (mm)	0.034	1	0.034	1.407	0.237	0.007
		Age group	0.130	4	0.032	1.337	0.258	0.026
		Error	4.773	197	0.024			

GC-IPLT = Ganglion cell-inner plexiform layer thickness, SCP VDs = Superficial capillary plexus vessel densities, DCP VDs = Deep capillary plexus vessel densities, SD = Standard deviation, SE = Standard error, AXL = Axial length, mm = Millimeter, G1 = Subjects aged 20–29 years, G2 = Subjects aged 30–39 years, G3 = Subjects aged 40–49 years, G4 = Subjects aged 50–59 years, and G5 = Subjects aged 60–69 years, µm = Micrometer, η^2^ = Eta Squared, % = Percentage, *n* = Number of subjects.

**Table 5:** Age-group comparison of FAZ and capillary flow parameters (*n* = 203).

Parameters	Data Analyses	Age Groups	Observed Mean ± SD		Adjusted Mean ± SE		Post-Hoc Analysis	
FAZ parameters								
FAZ area (mm^2^)	Descriptive statistics	G1	0.27 ± 0.09		0.27 ± 0.02			
		G2	0.26 ± 0.08		0.26 ± 0.02			
		G3	0.27 ± 0.14		0.27 ± 0.02			
		G4	0.28 ± 0.11		0.28 ± 0.02			
		G5	0.30 ± 0.08		0.315 ± 0.02			
			SS	DF	MS	F	*p* value	Partial η^2^
	Ancova results	AXL (mm)	0.018	1	0.018	1.743	0.188	0.009
		Age group	0.052	4	0.013	1.231	0.299	0.024
		Error	2.087	197	0.011			
FAZ perimeter (mm)	Descriptive statistics	G1	2.01 ± 0.34		2.01 ± 0.06			
		G2	1.93 ± 0.35		1.92 ± 0.06			
		G3	1.95 ± 0.50		1.95 ± 0.06			
		G4	2.02 ± 0.46		2.02 ± 0.06			
		G5	2.13 ± 0.30		2.14 ± 0.06			
			SS	DF	MS	F	*p* value	Partial η^2^
	Ancova results	AXL (mm)	0.042	1	0.042	0.267	0.606	0.001
		Age group	1.059	4	0.265	1.685	0.155	0.033
		Error	30.955	197	0.157			
FD-300 (%)	Descriptive statistics	G1	57.12 ± 3.88		57.06 ± 0.58		G1 > G5 (<0.001) G2 > G5 (0.011) G3 > G5 (0.037)	
		G2	56.46 ± 2.72		56.35 ± 0.59			
		G3	55.94 ± 4.24		55.97 ± 0.58			
		G4	55.63 ± 3.82		55.68 ± 0.59			
		G5	53.45 ± 3.70		53.54 ± 0.59			
			SS	DF	MS	F	*p* value	Partial η^2^
	Ancova results	AXL (mm)	12.355	1	12.355	0.899	0.344	0.005
		Age group	267.667	4	66.917	4.870	0.001 ***	0.090
		Error	2706.694	197	13.740			
Capillary flow parameters								
Outer retinal flow (mm^2^)	Descriptive statistics	G1	8.93 ± 2.00		8.94 ± 0.32			
		G2	8.25 ± 1.32		8.25 ± 0.32			
		G3	8.23 ± 1.96		8.23 ± 0.32			
		G4	8.92 ± 2.33		8.92 ± 0.32			
		G5	8.97 ± 2.33		8.96 ± 0.32			
			SS	DF	MS	F	*p* value	Partial η^2^
	Ancova results	AXL (mm)	0.077	1	0.077	0.019	0.891	0.000
		Age group	23.246	4	5.812	1.422	0.228	0.028
		Error	804.993	197	4.086			
Choriocapillaris flow (mm^2^)	Descriptive statistics	G1	20.38 ± 0.86		20.39 ± 0.15		G1> G2 (0.040) G1 > G3 (0.005) G1 > G4 (0.002) G1 > G5 (<0.001)	
		G2	19.75 ± 0.82		19.76 ± 0.16			
		G3	19.63 ± 0.84		19.62 ± 0.15			
		G4	19.57 ± 1.25		19.56 ± 0.16			
		G5	19.25 ± 1.02		19.24 ± 0.16			
			SS	DF	MS	F	*p* value	Partial η^2^
	Ancova results	AXL (mm)	0.193	1	0.193	0.203	0.652	0.001
		Age group	27.982	4	6.995	7.383	0.000 ***	0.130
		Error	186.671	197	0.948			

FAZ = Foveal avascular zone, FD-300 = Foveal VD in a 300-μm wide region surrounding the FAZ, SD = Standard deviation, SE = Standard error, AXL = Axial length, mm = millimeter, G1 = Subjects aged 20–29 years, G2 = subjects aged 30–39 years, G3 = Subjects aged 40–49 years, G4 = Subjects aged 50–59 years, and G5 = Subjects aged 60–69 years, µm = Micrometer, mm^2^ = Millimeter square, η^2^ = Eta Squared, % = Percentage, *** *p* < 0.001.

**Table 6:** Age-group comparative analysis of the ONH microstructural and microvascular parameters (*n* = 203).

Parameters	Data Analyses	Age Groups	Observed Mean ± SD		Adjusted Mean ± SE		Post-Hoc Analysis	
ONH microstructural parameter								
Global ONH RNFLT (µm)	Descriptive statistics	G1	118.78 ± 9.95		118.37 ± 1.89			
		G2	118.20 ± 13.62		117.47 ± 1.92			
		G3	112.78 ± 12.00		112.95 ± 1.88			
		G4	115.38 ± 10.44		115.75 ± 1.91			
		G5	114.75 ± 14.06		115.37 ± 1.93			
			SS	DF	MS	F	*p* value	Partial η^2^
	Ancova results	AXL (mm)	538.790	1	538.790	3.717	0.055	0.019
		Age group	691.885	4	172.971	1.193	0.315	0.024
		Error	28,552.573	197	144.937			
Cup-disc ratio	Descriptive statistics	G1	0.198 ± 0.082		0.198 ± 0.010			
		G2	0.199 ± 0.055		0.199 ± 0.011			
		G3	0.198 ± 0.073		0.198 ± 0.010			
		G4	0.206 ± 0.057		0.205 ± 0.011			
		G5	0.191 ± 0.060		0.191 ± 0.011			
			SS	DF	MS	F	*p* value	Partial η2
	Ancova results	AXL (mm)	8.706 × 10^−6^	1	8.706 × 10^−6^	0.002	0.965	0.000
		Age group	0.004	4	0.001	0.247	0.911	0.005
		Error	0.871	197	0.004			
ONH microvascular parameters								
wiVD (%)	Descriptive statistics	G1	49.43 ± 2.39		49.28 ± 0.43			
		G2	49.73 ± 2.79		49.44 ± 0.43			
		G3	49.69 ± 3.12		49.75 ± 0.43			
		G4	50.39 ± 2.42		50.54 ± 0.43			
		G5	48.99 ± 3.13		49.23 ± 0.44			
			SS	DF	MS	F	*p* value	Partial η2
	Ancova results	AXL (mm)	81.319	1	81.319	10.984	0.001 ***	0.053
		Age group	46.090	4	11.523	1.556	0.188	0.031
		Error	1458.477	197	7.403			
Inside disc VDs (%)	Descriptive statistics	G1	52.82 ± 3.74		52.89 ± 0.62			
		G2	53.48 ± 4.15		53.59 ± 0.63			
		G3	51.97 ± 4.49		51.94 ± 0.62			
		G4	52.45 ± 3.69		52.39 ± 0.63			
		G5	51.68 ± 3.67		51.58 ± 0.64			
			SS	DF	MS	F	*p* value	Partial η^2^
	Ancova results	AXL (mm)	12.261	1	12.261	0.779	0.378	0.004
		Age group	94.208	4	23.552	1.497	0.204	0.030
		Error	3098.834	197	15.730			
Peripapillary VDs (%)	Descriptive statistics	G1	51.60 ± 2.82		51.46 ± 0.51			
		G2	52.36 ± 3.18		52.12 ± 0.51			
		G3	52.37 ± 3.69		52.43 ± 0.50			
		G4	52.91 ± 2.95		53.04 ± 0.51			
		G5	51.82 ± 3.55		52.03 ± 0.52			
			SS	DF	MS	F	*p* value	Partial η^2^
	Ancova results	AXL (mm)	62.501	1	62.501	6.048	0.015 *	0.030
		Age group	52.908	4	13.227	1.280	0.279	0.025
		Error	2035.879	197	10.334			

ONH = Optic nerve head, RNFLT = Retinal nerve fiber layer thickness, VDs = Vessel densities, SD = Standard deviation, SE = Standard error, AXL = Axial length, mm = millimeter, G1 = Subjects aged 20–29 years, G2 = subjects aged 30–39 years, G3 = Subjects aged 40–49 years, G4 = Subjects aged 50–59 years, and G5 = Subjects aged 60–69 years, µm = Micrometer, η^2^ = Eta Squared, % = Percentage, * *p* < 0.05, *** *p* < 0.001.

## Data Availability

The manuscript contains all data. The datasets used and/or analyzed in this study, however, are available upon reasonable request from a corresponding author.
